# Alcohol Consumption Among Young Adults Ages 18–24 in the United States: Results from the 2001–2002 NESARC Survey

**Published:** 2004

**Authors:** Chiung M. Chen, Mary C. Dufour, Hsiao-ye Yi

**Affiliations:** Chiung M. Chen, M.A., is an analyst; Mary C. Dufour, M.D., M.P.H., is a senior associate; and Hsiao-ye Yi, Ph.D., is a senior analyst, all with the Alcohol Epidemiologic Data System of the National Institute on Alcohol Abuse and Alcoholism (NIAAA), which is operated by CSR, Incorporated, Arlington, Virginia

**Keywords:** young adult, undergraduate student, underage drinking, National Epidemiologic Survey on Alcohol and Related Conditions (NESARC), heavy drinking, binge drinking, AOD (alcohol and other drug) use pattern, AOD use frequency, amount of AOD use, AOD intake per occasion, AOD use frequency, individual AOD consumption, aggregate AOD consumption, gender differences, ethnic differences, racial differences

## Abstract

The high prevalence of drinking in young adults is a serious public health concern. Alcohol use among young adults often is associated with a wide variety of risky behaviors and both immediate and long-term negative consequences. The 2001–2002 National Epidemiologic Survey on Alcohol and Related Conditions (NESARC) presents a unique opportunity to examine young adult drinking because it has an excellent response rate, oversamples young adults ages 18–24, and includes college-related group housing. According to the NESARC data, in 2001–2002 over three-quarters of young adults ages 21–24 were current drinkers, as were nearly two-thirds of those ages 18–20, despite the fact that the legal drinking age is 21. More than half of young adult men exceeded the recommended daily drinking limit, as did two-fifths of young adult women. Although the prevalence of exceeding the daily limit is higher for those ages 21–24 than for those ages 18–20, it also is substantial for those ages 18–20. Because drinking more than the recommended per-occasion maximum is likely to impair mental and physical performance, the increase over the past decade in the prevalence among young adults of drinking five or more drinks 12 or more times per year may help explain the increased risk of injury and other acute negative consequences commonly observed among college students ages 18–24.

Over the life course, drinkers in the United States tend to have the highest level of alcohol consumption in their late teens and early twenties (Naimi et al. 2003; Fillmore et al. 1991). According to the National Survey on Drug Use and Health (NSDUH), the prevalences of both binge drinking (i.e., consuming five or more [5+] drinks in a row at least once in the past month) and heavy drinking (i.e., consuming 5+ drinks in a row on at least five occasions in the past month) in 2003 were highest among young adults ages 18 to 25, peaking at age 21 (Substance Abuse and Mental Health Services Administration [SAMHSA] 2004). And according to the Monitoring the Future Survey, the prevalence of drinking and heavy drinking (i.e., consuming 5+ drinks in a row at least once in the prior 2-week period) among young adults changed relatively little between 1993 and 2003 after declining noticeably from 1980 to 1993 (Johnston et al. 2004).

The high prevalence of drinking in young adults is a serious public health concern because alcohol use by this age group often is associated with a wide variety of risky behaviors and various negative consequences. Many of these consequences are immediate and tragic (Hingson et al. 2005)—most notably alcohol-related traffic fatalities (Yi et al. 2004).

College students continue to stand out from other young adults because of their relatively high rates of heavy drinking, even though their average daily alcohol consumption generally is lower than that of their noncollege peers (Johnston et al. 2004). Until recently, however, college students have been a difficult population to study. In general, they are not well represented in normal household surveys, which typically exclude group housing, such as dormitories, fraternities, and sororities. In addition, group-housing-based samples of college students must be quite large in order to attain accurate national representation because there is great heterogeneity in the types of student populations served in these institutions. Finally, the drinking behavior of young adults, particularly college students, often is characterized by episodic drinking, which may be more difficult to capture adequately on surveys that rely only on the measure of average alcohol consumption over a short period of time.

This Epidemiological Bulletin draws on the rich data on young adult drinking behavior available from the 2001–2002 National Epidemiologic Survey on Alcohol and Related Conditions (NESARC). NESARC is uniquely suited to examine young adult drinking for three reasons—the excellent response rate, the oversampling of young adults ages 18–24, and the inclusion of college-related group housing. Findings presented here include the prevalence of alcohol use in youth ages 18–24; drinking frequency and quantity; frequency of heavy drinking, intoxication, and driving after drinking; as well as age of drinking onset and choice of drinking locations for different types of alcoholic beverages. In particular, this Bulletin examines the number of young adults who exceed daily and weekly guidelines for low-risk drinking. The current public health recommendations for the nation—*Healthy People 2010* (Department of Health and Human Services 2000)—include a number of goals related to alcohol use. Recognizing the importance of both drinking volume and pattern of consumption as predictors of alcohol-related harm, these recommendations include, for the first time, an explicit objective to “reduce the proportion of adults who exceed guidelines for low-risk drinking” (Objective 26–13). The accompanying text explains that men may be at risk for alcohol-related problems if they drink more than 14 drinks per week or more than 4 drinks per occasion and that women may be at risk if they drink more than 7 drinks per week or more than 3 drinks per occasion. Most people who exceed these guidelines do so by drinking more than the specified maximum number of drinks per occasion at least once a year. Drinking more than the per-occasion maximum impairs mental performance and physical coordination, increasing the risk of injury.

In addition, this Bulletin provides insight into the drinking behavior of subgroups of young adults based on age, sex, race/ethnicity, and college enrollment status. For example, the definition of young adults conventionally includes all people ages 18–24. Yet people who fall into the younger part of this group (i.e., those ages 18–20) are below the legal drinking age of 21. Underage drinking remains a major public health concern. To better understand the scope of this problem, the data presented here are given for the total young adult population as well as for the subgroups of people ages 18–20 and 21–24. Finally, recent trends in young adult risk drinking are briefly examined by comparing the NESARC results with those from the 1991–1992 National Longitudinal Alcohol Epidemiologic Survey (NLAES).

## Methods

### Data

The analyses presented here are based on data from the 2001–2002 NESARC, which was designed and sponsored by the National Institute on Alcohol Abuse and Alcoholism (NIAAA) and fielded by the U.S. Bureau of the Census. Data were collected in face-to-face, computer-assisted personal interviews that were conducted in respondents’ homes. The NESARC sample represents the civilian, noninstitutionalized adult population of the United States, including residents of all 50 States and the District of Columbia (Grant et al. 2003). It includes people living in households, military personnel living off base, and people living in the following group quarters: boarding or rooming houses, nontransient hotels and motels, shelters, facilities for housing workers, college quarters, and group homes. The sampling frames for housing units and group quarter units are derived from the Census 2000/2001 Supplementary Survey and the Census 2000 Group Quarters Inventory, respectively. One sample adult was selected for interview in each household. NESARC oversampled Black and Hispanic households and adults ages 18–24 to obtain more reliable estimates for these groups. The sampling frame response rate was 99 percent, the household response rate was 89 percent, and the person response rate was 93 percent, yielding an overall response rate of 81 percent.

A total of 43,093 respondents age 18 and older completed the survey. The data reported here are based on responses from 5,199 young adults ages 18–24, including 3,551 current drinkers (i.e., young adults who reported drinking at least one alcoholic drink in the 12 months prior to the survey).

### Drinking Measures

NESARC measures alcohol consumption separately for four specific types of alcoholic beverages (i.e., coolers, beer, wine, and liquor), detailing for each beverage the usual and heaviest quantities and frequencies, as well as the usual time and place of drinking. Nevertheless, the variables selected to represent the current drinking status of young adults pertain to any alcohol use regardless of beverage type. These items were taken directly from the survey and had a reference period of the last 12 months. The only exception is the daily volume of alcohol (i.e., ethanol) consumption. This measure was derived by NIAAA using a somewhat complex algorithm that summed beverage-specific volumes across the four beverage types.^1^ In NESARC, one standard drink is assumed to contain 0.6 ounces of ethanol. The various drinking measures were defined as follows:

- *Current drinking*—Had at least one drink of alcohol in the past year.
- *Drinking frequency*—Number of days on which any alcoholic beverages were consumed in the past year.^2^
- *Drinking quantity*—Usual number of drinks (all types of alcohol combined) consumed on drinking days in the past year.
- *Risk drinking*—Drinking in excess of recommended drinking limits (for more detailed definitions, see the next paragraph).
- *Intoxication*—Drinking enough to feel intoxicated or drunk (e.g., exhibiting slurred speech or blurred vision or feeling unsteady on one’s feet).
- *Tolerance*—Reported maximum number of drinks the respondent can hold without feeling intoxicated or drunk.
- *Driving after drinking*—Driving a car or other motor vehicle (e.g., motorcycle, boat, jet ski, or skimobile) after consuming three or more drinks on one occasion in the past year.
- *Drinking location for beverage type*—Usual location of drinking for each type of alcoholic beverage (i.e., coolers, beer, wine, and liquor), including own home, home of friends or relatives, or public places (such as a bar, restaurant, or sports arena).
- *Age of drinking onset*—Age when the respondent first started drinking (not counting small tastes or sips of alcohol).

For risk drinking, the following subcategories were specified:

- *Exceeding the weekly drinking limit*—On average, consuming more than two drinks (1.2 ounces of ethanol) per day for men and more than one drink (0.6 ounces of ethanol) per day for women in the past year.
- *Exceeding the daily drinking limit*—Consuming more than four drinks (2.4 ounces of ethanol) in a single day for men and more than three drinks (1.8 ounces of ethanol) in a single day for women one or more times in the past year.
- *Episodic heavy drinking*—Consuming 5+ drinks for men and four or more drinks for women in a single day in the past year.

### Data Analysis

Data presented in this article are descriptive statistics. The estimates of the drinking measures for the various subpopulations are crude values and are not adjusted for other variables. Percentages of drinking and risk drinking were calculated for the total young adult population, whereas drinking behaviors—including frequencies of drinking, episodic heavy drinking, intoxication, and driving after drinking 3+ drinks—as well as drinking quantities and tolerance were calculated for current drinkers. Both for the total young adult sample and for the two age subgroups, analyses were carried out by sex, race/ethnicity (i.e., White, Black, American Indian/Alaska Native [AIAN], Asian/Native Hawaiian and Other Pacific Islander [NHOPI], and Hispanic of all races), and college enrollment status (i.e., full-time college students, part-time college students, and noncollege peers).

**Figure 1:**
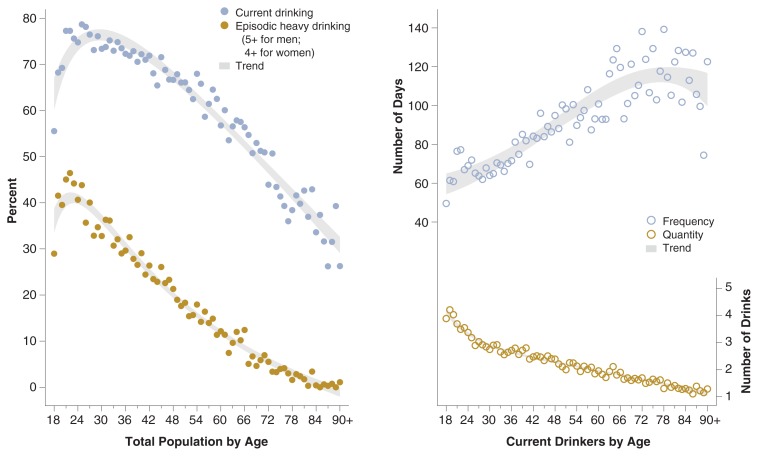
Percentages of current drinking and episodic heavy drinking by age (graph on the left), and mean frequency and quantity of drinking by age (graph on the right), NESARC, 2001–2002.

All estimates were weighted by the sampling weights to represent the entire U.S. young adult population. Standard errors of estimates were produced by the software package SUDAAN, which takes into account the complex sampling design employed by NESARC using the Taylor series linearization method (Research Triangle Institute 2004). In figure 1, age trends are represented by the 95-percent confidence limits of predicted means based on fractional polynomial regression using age as the covariate. This procedure increases the flexibility of conventional polynomial models by finding the best-fitting powers of continuous covariates (Royston and Altman 1994).

## Results

In order to put young adult drinking in context, figure 1 displays the prevalence of current drinking and episodic heavy drinking as well as mean drinking frequency and quantity by age for the total U.S. population age 18 and older. The trend for current drinking increases with age in the late teens and early twenties, reaches its peak around age 25 or 26, and decreases thereafter. The trend for episodic heavy drinking shows a similar pattern, but peaks a few years earlier, around age 21 or 22. Among current drinkers, the average drinking quantity declines almost linearly with age, whereas the average drinking frequency shows an upward trend through the mid-seventies.

### Prevalence of Drinking and Risk Drinking Among Young Adults

**Figure 2:**
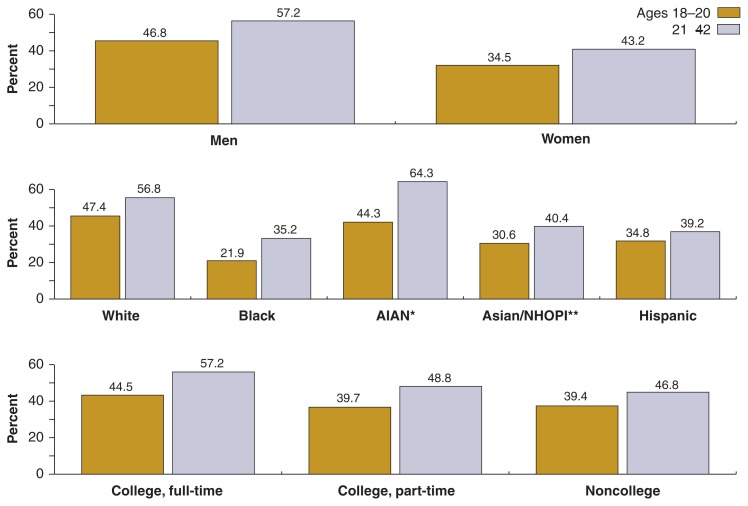
Percentages of exceeding the daily drinking limit for ages 18–20 and 21–24, by sex, race/ethnicity, and college enrollment status, NESARC, 2001–2002. ** NHOPI = Native Hawaiian/Other Pacific Islander * AIAN = American Indian/Alaska Native

**Figure 3:**
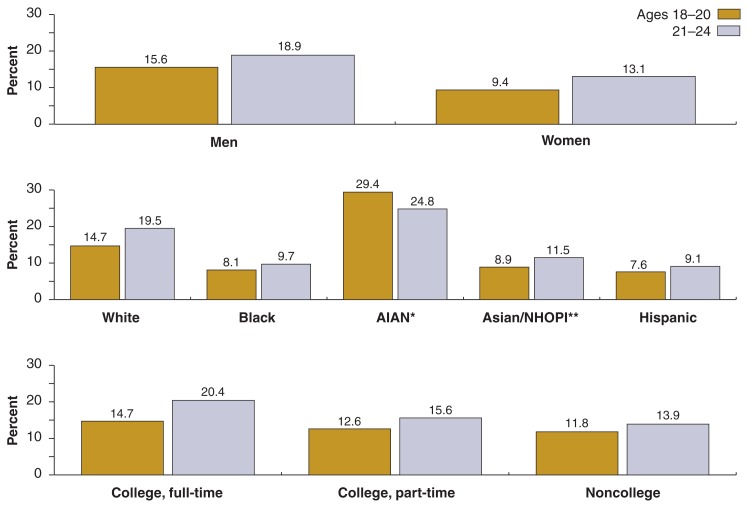
Percentages of exceeding the weekly drinking limit for ages 18–20 and 21–24, by sex, race/ethnicity, and college enrollment status, NESARC, 2001–2002. * AIAN = American Indian/Alaska Native **NHOPI = Native Hawaiian/Other Pacific Islander

Table 1 presents the estimated prevalence of drinking and risk drinking among young adults by sex, race/ethnicity, and college enrollment status, both for the total population and for the two age groups 18–20 and 21–24. Data for the two age groups also are presented graphically in figures 2 and 3.

According to these data, in 2001–2002 about 70 percent of young adults in the United States (or about 19 million) consumed alcohol in the year prior to the survey. Alcohol consumption was more common among males, with three-quarters of young adult men (about 10 million) reporting drinking in the past year, compared with two-thirds of young adult women (about 9 million).

Risk drinking also was common among young adults. About 46 percent (12.4 million) of young adults engaged at least once in drinking that exceeded the recommended daily limits and 14.5 percent (3.9 million) had an average consumption that exceeded the recommended weekly limits.

#### Differences Between Age Groups

Drinking was more common among the older subgroup of young adults, with more than three-quarters of young adults ages 21–24 reporting drinking in the past year, compared with slightly less than two-thirds of those ages 18–20. The prevalence of exceeding the daily and weekly limits also was generally higher among young adults ages 21–24 (50.0 percent and 16.0 percent, respectively) than among those ages 18–20 (40.8 percent and 12.6 percent, respectively). This was true for all breakdowns by sex, race/ethnicity (with the exception of AIAN), and college enrollment status, although some of the differences were not statistically significant^3^ (see figures 2 and 3).

#### Gender Differences

Substantial gender differences existed in the drinking behavior of young adults (see table 1). More than half of young men (7.1 million) exceeded the recommended daily limit compared with two-fifths of the young women (5.3 million). Similarly, one-sixth (17.4 percent or 2.4 million) of young men exceeded the recommended weekly limit compared with one-ninth of young women (11.5 percent or 1.6 million).

#### Influence of Race/Ethnicity

Among the racial/ethnic groups, prevalence of drinking was higher for Whites than for other groups in 2001–2002. An estimated 77.1 percent of Whites were classified as current drinkers, compared with 70.7 percent of AIAN, 60.1 percent of Blacks, 60.4 percent of Hispanics, and 59.1 percent of Asians/NHOPI. The prevalence of exceeding the recommended daily and weekly drinking limits also was higher for Whites (52.5 percent and 17.3 percent, respectively) and AIAN (53.0 and 27.4 percent) than for Asians/NHOPI (36.5 percent and 10.5 percent), Hispanics (37.3 percent and 8.5 percent), and Blacks (29.0 percent and 8.9 percent).

#### Influence of College Enrollment Status

Differences also existed with respect to college enrollment status. College students had a higher prevalence of drinking and risk drinking than did their noncollege peers. Thus, 74.9 percent of fulltime college students and 76.3 percent of part-time college students drank alcohol during the past 12 months, compared with 68.3 percent of their noncollege peers. Full-time college students also had the highest percentage of exceeding the recommended daily and weekly limits (51.7 percent and 17.9 percent, respectively), followed by part-time college students (45.9 percent and 14.6 percent), and noncollege peers (43.3 percent and 12.9 percent).

### Specific Drinking Behaviors Among Young Adult Drinkers

Table 2 summarizes data on different patterns of drinking, episodic heavy drinking, and related behaviors among current drinkers classified by sex, race/ethnicity, and college enrollment status for the total young adult population and for the two age groups. These patterns are compared in terms of the mean for each of the population subgroups.

On average, young adult drinkers consumed alcohol on about 66 days per year. On the days that they drank, respondents reported engaging in episodic heavy drinking on 39 days, drinking enough to be intoxicated on 18 days, and driving after drinking 3 or more drinks on approximately 5 days. The respondents’ average drinking quantity was 3.7 drinks per drinking day, and the reported tolerance was 4.2 drinks. The values for all these measures were more than 50–90 percent higher for young men than for young women.

**Figure 4:**
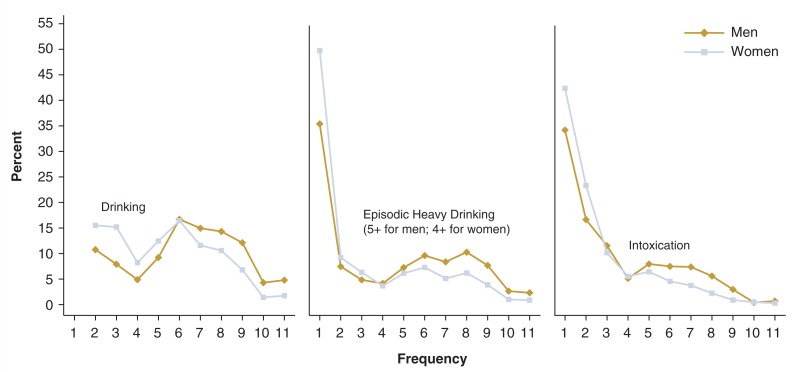
Frequency of drinking, episodic heavy drinking, and intoxication among current drinkers ages 18–24, by sex, NESARC, 2001–2002, as follows: 1 = None 2 = 1–2 times in the last year 3 = 3–6 times in the last year 4 = 7–11 times in the last year 5 = Once a month 6 = 2–3 times a month 7 = Once a week 8 = 2 times a week 9 = 3–4 times a week 10 = Nearly every day 11 = Every day

**Figure 5:**
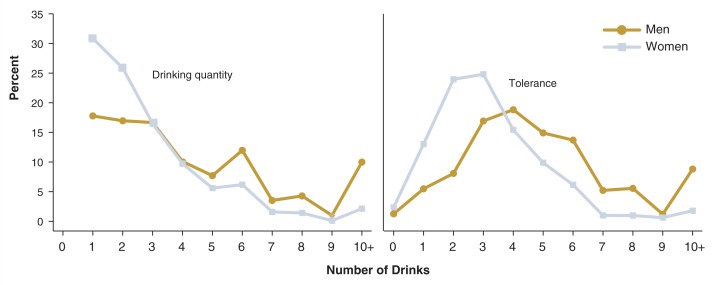
Drinking quantity* and tolerance* among current drinkers ages 18–24, by sex, NESARC, 2001–2002. *See text, p. 270, for definitions of terms.

Figures 4 and 5 show detailed distributions of these drinking measures for drinkers ages 18–24. Most of the distributions were positively skewed, indicating that the mean values of these measures were inflated because a small proportion of drinkers reported very high values. For instance, the average drinking quantity for young adult male drinkers was 4.5 drinks per drinking day. As shown in figure 5, however, more than half of these drinkers consumed 1–3 drinks per drinking day, whereas approximately 10 percent reported having 10 or more drinks per drinking day. Similarly, the average frequency of intoxication among young adult male drinkers was about twice a month (23.3 days per year), whereas more than 65 percent of these drinkers reported being intoxicated less than once per month (see figure 4). The seemingly high average frequency resulted from the fact that a small proportion (about 10 percent) of the drinkers reported being intoxicated twice a week or more.

#### Differences Between Age Groups

Comparison of young adults ages 18–20 with those ages 21–24 found some differences between the two age groups. People in the older age group had a higher frequency of drinking and episodic heavy drinking (72.5 and 39.1 days per year, respectively) compared with people in the younger age group (57.7 and 38.3 days per year, respectively). In contrast, young adults ages 18–20 had a higher frequency of intoxication (20.0 days) and driving after drinking three or more drinks (5.1 days), higher drinking quantity (4.1 drinks), and higher tolerance (4.3 drinks) than those ages 21–24.

#### Influence of Race/Ethnicity

Among the racial/ethnic groups, AIAN young adult drinkers had the youngest average age of drinking onset (15.4 years); the highest estimates for frequency of drinking (97.2 days), episodic heavy drinking (60.2 days), and intoxication (39.8 days); and the highest drinking quantity (5.4 drinks/drinking day).^4^ In contrast, Black young adult drinkers had the oldest average age of drinking onset (18 years), the lowest estimates for frequency of episodic heavy drinking (22.9 days) and intoxication (13.2 days), and the lowest drinking quantity (2.7 drinks/drinking day) and tolerance (3.7 drinks).

#### Influence of College Enrollment Status

With regard to college enrollment status, full-time college student drinkers appeared to have the highest estimates for frequencies of drinking (71.6 days), episodic heavy drinking (44.3 days), intoxication (21.1 days), and driving after drinking three or more drinks (6.2 days), whereas part-time college student drinkers had the lowest estimates and their noncollege peers had intermediate estimates for these measures. For drinking quantity and tolerance, noncollege drinkers reported slightly higher averages (3.8 and 4.4 drinks, respectively) than the two college groups. It is important to note that because of some relatively large standard errors, not all differences among the three groups may be statistically significant.

### Usual Drinking Locations

Table 3 summarizes the distribution of beverage-specific drinking locations for the two age groups by sex, race/ethnicity, and college enrollment status. With a few exceptions related to wine consumption, young adults ages 18–20 were more likely to report drinking in the homes of friends and relatives (59.1 percent for coolers, 62 percent for beer, 43 percent for wine, and 61.8 percent for liquor) than in their own homes or in public places. In contrast, the drinking locations of people ages 21–24 varied by type of alcoholic beverage. For instance, 57.1 percent of drinkers ages 21–24 chose public places to consume liquor, whereas wine and coolers were most commonly consumed in the respondents’ own homes (39 and 37.8 percent, respectively).

The choice of drinking location for beer varied depending on sex, race/ethnicity, and college enrollment status. For example, among young adults ages 21–24, men appeared most likely to choose their own homes (41.2 percent), whereas women were most likely to choose public places (50.7 percent) to consume beer. Blacks and Hispanics were most likely to choose their own homes (41.5 percent and 51.4 percent, respectively) to consume beer, whereas Whites chose public places (43 percent). Differences in drinking locations for beer also were observed between college students and their noncollege peers in the 21–24 age group, with college students being more likely to drink in public places.

### Changes in Risk Drinking Over the Past Decade

**Figure 6:**
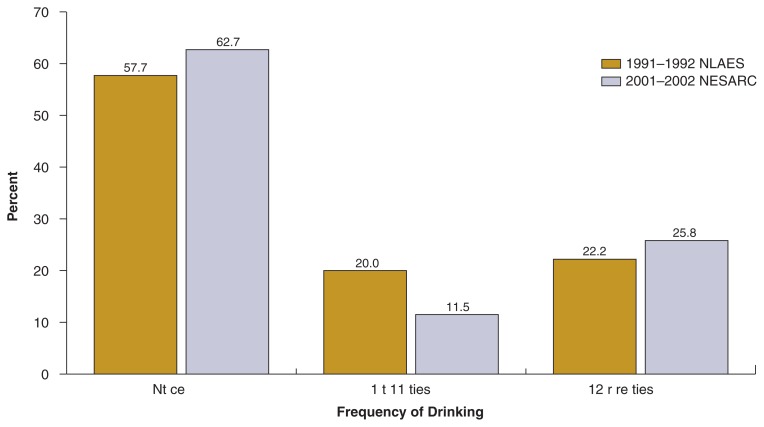
Percent distribution of frequency of drinking 5+ drinks in a single day in the past year, among young adults ages 18–24 in the United States.

Despite slight differences in the survey designs, comparing data from the 2001–2002 NESARC with that of its predecessor, the 1991–1992 National Longitudinal Alcohol Epidemiologic Survey (NLAES; also designed by NIAAA), provides valuable insight into the trends in young adult drinking over the past decade. One comparable measure of risk drinking in the two surveys—the frequency of drinking 5+ drinks on a single day—was selected to illustrate the changes (see figure 6).

The comparison shows that during the 10 years between the two surveys, some progress was made in reducing episodic heavy drinking among young adults ages 18–24. In 2001–2002, 62.7 percent of young adults in this age group did not engage in episodic heavy drinking, compared with 57.7 percent in 1991–1992 (NIAAA 1998). Among those who already were engaged in such risk drinking, however, there was a shift toward higher frequencies of heavy episodic drinking. Although the percentage of young adults drinking 5+ drinks 1 to 11 times in the past year declined by nearly half, the percentage of those drinking this amount 12 or more times in the past year increased from 22.2 percent in 1991–1992 (NIAAA 1998) to 25.8 percent in 2001–2002.

## Discussion

This Epidemiological Bulletin provides a broad overview of the unique consumption patterns and hazardous nature of young adult drinking in the United States using data from NESARC, NIAAA’s most comprehensive and up-to-date national survey. According to the NESARC data, over three-quarters of young adults ages 21–24 were current drinkers in 2001–2002. Interestingly, nearly two-thirds of 18- through 20-year-olds were current drinkers, despite the fact that the legal drinking age is 21. More than half of young adult men exceeded the recommended daily drinking limit, as did two-fifths of young adult women. Although the prevalence of exceeding the daily limit is higher for those ages 21–24 than for those ages 18–20, the prevalence for those ages 18–20 is still substantial.

A comparison of NESARC and NLAES indicates a trend toward a higher frequency of 5+ drinking for young adults who engaged in 5+ drinking in NESARC. The National Alcohol Survey (Greenfield et al. 2003) found a similar trend for underage drinkers ages 18–20 between 1995 and 2000; during this period the weekly heavy drinkers doubled their rate of 5+ drinking, with a significant increase seen for those drinking 5+ drinks more than weekly (Greenfield et al. 2003). When examining the trend of college binge drinking, the Harvard School of Public Health College Alcohol Study also found that the percentages of both abstainers and frequent binge drinkers increased during the period from 1993 to 2001 (Wechsler et al. 2002). Because drinking more than the recommended per-occasion maximum is likely to impair mental and physical performance, the increase in the prevalence of consuming 5+ drinks 12 or more times per year that has been observed over the past decade may help explain the increased risk of injury and other acute negative consequences found among college students ages 18–24 (Hingson et al. 2005).

It should be stressed, however, that the estimates for population subgroups are descriptive in nature and are not adjusted for possible confounding factors. Therefore, caution should be taken when interpreting the results or making causal inferences based on patterns observed in this study.

Although comparisons with other national surveys will invariably yield more informed conclusions, interviewer training, respondents’ interpretation of questions, and other differing survey processes may contribute to the discrepancies between NESARC and other national surveys. For example, the prevalence estimate of past-year alcohol use among young adults ages 18–24 from the 2001–2002 NESARC is 70.8 percent (74.7 percent for males and 66.8 percent for females). Two other national surveys that allow for prevalence estimates of past-year alcohol use among young adults are NSDUH^5^ and the National Health Interview Survey (NHIS). Based on our calculation, the 2001 and 2002 NSDUH on average yield a much higher prevalence of past-year alcohol use—76.6 percent (79.2 percent for males and 74.0 percent for females)—among the 18–25 age group than does NESARC. Only adding young adults age 25 is not likely to account for the discrepancy between these two sets of estimates. The 2001 and 2002 NHIS surveys, on the other hand, provide a much lower estimate—63.9 percent (70.1 percent for males and 57.7 percent for females)—than NESARC. Further studies are needed to investigate the sources of these discrepancies between NESARC and other national surveys.

Early initiation of alcohol use has been found to be positively associated with alcohol abuse and dependence as well as other alcohol-related problems (e.g., risky patterns of consumption) (Grant and Dawson 1997; Grant et al. 2001). Therefore, in the long term, developing and implementing effective prevention strategies targeted toward adolescents may be one key to reducing young adult drinking, especially underage drinking and risky patterns of consumption. Indeed, community-based interventions have shown promise in reducing high-risk drinking and other alcohol-related outcomes among underage drinkers (Wagenaar et al. 2000; Holder et al. 2000) and may be effective with young adults. Other approaches to reducing underage drinking have been summarized by the Institute of Medicine (National Research Council and Institute of Medicine 2004). All of these measures, however, will require some time until they can be fully developed and/or implemented. In the interim, steps need to be taken to increase public awareness of the *Healthy People 2010* low-risk drinking guidelines, particularly among young adults.

## Figures and Tables

**Table 1:** Prevalence of Drinking, Exceeding the Daily Drinking Limit, and Exceeding the Weekly Drinking Limit in the Past Year Among the Total Population of Young Adults Ages 18–20 and 21–24, According to Sex, Race/Ethnicity, and College Enrollment Status, NESARC, 2001–2002

Ages	Sex, Race/Ethnicity, College Enrollment Status	Past-Year Drinking		Exceeding Daily Drinking Limit		Exceeding Weekly Drinking Limit	
		
		%	(SE)^1^	%	(SE)	%	(SE)
**18–24**	**Total**	70.8	(1.0)	45.9	(1.1)	14.5	(0.7)
	Men	74.7	(1.2)	52.3	(1.4)	17.4	(1.0)
	Women	66.8	(1.6)	39.4	(1.5)	11.5	(0.8)
	White	77.1	(1.3)	52.5	(1.4)	17.3	(0.9)
	Black	60.1	(1.9)	29.0	(1.9)	8.9	(1.2)
	AIAN^2^	70.7	(6.4)	53.0	(6.8)	27.4	(6.2)
	Asian/NHOPI^3^	59.1	(4.3)	36.5	(4.0)	10.5	(2.3)
	Hispanic	60.4	(1.8)	37.3	(2.0)	8.5	(0.9)
	College, full-time	74.9	(1.5)	51.7	(1.8)	17.9	(1.5)
	College, part-time	76.3	(2.5)	45.9	(3.1)	14.6	(2.2)
	Noncollege	68.3	(1.2)	43.3	(1.2)	12.9	(0.7)
**18–20**	**Total**	64.2	(1.5)	40.8	(1.7)	12.6	(1.0)
	Men	68.4	(1.7)	46.8	(2.1)	15.6	(1.5)
	Women	59.7	(2.2)	34.5	(2.2)	9.4	(1.2)
	White	70.1	(1.9)	47.4	(2.1)	14.7	(1.3)
	Black	49.1	(2.6)	21.9	(2.2)	8.1	(1.5)
	AIAN	64.9	(9.2)	44.3	(9.3)	29.4	(8.7)
	Asian/NHOPI	55.2	(6.4)	30.6	(6.1)	8.9	(4.5)
	Hispanic	57.4	(3.0)	34.8	(3.5)	7.6	(1.4)
	College, full-time	68.4	(2.5)	44.5	(2.6)	14.7	(1.9)
	College, part-time	65.4	(5.3)	39.7	(5.8)	12.6	(4.5)
	Noncollege	62.4	(1.7)	39.4	(1.9)	11.8	(1.0)
**21–24**	**Total**	76.2	(1.2)	50.0	(1.3)	16.0	(0.9)
	Men	80.3	(1.6)	57.2	(1.8)	18.9	(1.4)
	Women	72.4	(1.7)	43.2	(1.6)	13.1	(1.0)
	White	82.9	(1.5)	56.8	(1.7)	19.5	(1.2)
	Black	69.9	(2.6)	35.3	(3.0)	9.7	(1.6)
	AIAN	78.1	(7.9)	64.3	(8.6)	24.8	(8.3)
	Asian/NHOPI	61.6	(5.1)	40.4	(5.1)	11.5	(2.6)
	Hispanic	62.7	(2.5)	39.2	(2.7)	9.1	(1.3)
	College, full-time	79.9	(1.8)	57.2	(2.3)	20.4	(2.0)
	College, part-time	81.6	(2.6)	48.8	(3.7)	15.6	(2.7)
	Noncollege	73.6	(1.5)	46.8	(1.4)	13.9	(0.9)

1 SE = standard error

2 AIAN = American Indian/Alaska Native

3 NHOPI = Native Hawaiian/Other Pacific Islander

**Table 2:** Average Age of Drinking Onset, Drinking Frequency, Frequency of Episodic Heavy Drinking and Intoxication, Drinking Quantity, Quantity Held Without Becoming Intoxicated (Tolerance), and Frequency of Driving After Drinking 3+ Drinks in the Past Year Among Young Adult Current Drinkers Ages 18–20 and 21–24, According to Sex, Race/Ethnicity, and College Enrollment Status, NESARC, 2001–2002

Ages	Sex, Race/Ethnicity, College Enrollment Status	Age of Drinking Onset^1^		Drinking Frequency		Episodic Heavy Drinking		Intoxication		Drinking Quantity		Tolerance		Driving After Drinking 3+ Drinks	
						
		Year	(SE)^2^	Days	(SE)	Days	(SE)	Days	(SE)	Drink	(SE)	Drink	(SE)	Days	(SE)
**18–24**	**Total**	17.4	(0.0)	66.4	(1.7)	39.0	(1.6)	18.0	(1.0)	3.7	(0.1)	4.2	(0.1)	4.8	(0.6)
	Men	17.2	(0.1)	82.7	(2.8)	50.2	(2.6)	23.3	(1.4)	4.5	(0.1)	5.1	(0.1)	6.0	(0.9)
	Women	17.6	(0.1)	48.3	(2.0)	26.4	(1.8)	11.9	(1.2)	2.9	(0.1)	3.3	(0.1)	3.5	(0.7)
	White	17.4	(0.1)	69.7	(2.2)	43.0	(2.0)	18.9	(1.2)	3.9	(0.1)	4.2	(0.1)	5.1	(0.8)
	Black	18.0	(0.1)	64.7	(4.4)	22.9	(2.9)	13.2	(2.4)	2.7	(0.2)	3.7	(0.2)	2.6	(0.8)
	AIAN^3^	15.4	(0.6)	97.2	(17.2)	60.2	(16.3)	39.8	(12.9)	5.4	(0.7)	4.0	(0.3)	1.8	(0.8)
	Asian/NHOPI^4^	17.5	(0.2)	54.4	(6.7)	33.3	(6.3)	22.3	(6.8)	3.3	(0.3)	4.3	(0.4)	4.0	(1.7)
	Hispanic	17.6	(0.1)	53.0	(3.5)	32.0	(3.1)	13.4	(2.3)	3.8	(0.2)	4.9	(0.2)	5.8	(1.6)
	College, full-time	17.6	(0.1)	71.8	(3.2)	44.3	(2.9)	21.1	(1.8)	3.7	(0.1)	4.0	(0.1)	6.2	(1.4)
	College, part-time	17.8	(0.2)	55.8	(4.9)	30.7	(4.0)	15.7	(2.6)	3.5	(0.2)	4.1	(0.2)	2.5	(0.8)
	Noncollege	17.3	(0.1)	65.3	(2.0)	37.4	(1.8)	16.7	(1.2)	3.8	(0.1)	4.4	(0.1)	4.5	(0.6)
**18–20**	**Total**	16.7	(0.1)	57.7	(2.8)	38.8	(2.4)	20.0	(1.7)	4.1	(0.1)	4.3	(0.1)	5.1	(1.2)
	Men	16.6	(0.1)	70.6	(4.0)	49.1	(3.6)	24.4	(2.2)	5.0	(0.2)	5.1	(0.2)	7.7	(2.0)
	Women	16.8	(0.1)	42.0	(3.5)	26.3	(3.2)	14.6	(2.4)	3.0	(0.1)	3.2	(0.1)	2.0	(0.9)
	White	16.6	(0.1)	59.3	(3.6)	42.4	(3.3)	21.4	(2.2)	4.3	(0.2)	4.2	(0.1)	5.2	(1.5)
	Black	17.0	(0.1)	58.6	(7.3)	21.9	(5.9)	15.1	(4.1)	2.8	(0.4)	3.4	(0.4)	2.8	(1.6)
	AIAN	15.9	(0.6)	119.0	(26.1)	75.1	(25.2)	39.4	(17.9)	5.1	(0.9)	4.3	(0.5)	0.6	(0.5)
	Asian/NHOPI	17.0	(0.3)	49.2	(13.5)	38.4	(14.0)	33.2	(13.5)	3.7	(0.6)	4.4	(0.5)	0.2	(0.1)
	Hispanic	16.7	(0.2)	43.0	(4.6)	29.2	(3.8)	10.7	(1.8)	4.0	(0.3)	4.9	(0.3)	8.3	(3.4)
	College, full-time	17.2	(0.1)	59.9	(5.0)	39.7	(4.3)	20.9	(2.9)	3.9	(0.2)	4.0	(0.2)	8.4	(3.2)
	College, part-time	16.8	(0.3)	43.4	(8.6)	24.0	(7.0)	14.9	(5.9)	3.5	(0.5)	3.9	(0.3)	0.7	(0.3)
	Noncollege	16.4	(0.1)	58.0	(3.2)	39.6	(3.1)	20.0	(2.1)	4.2	(0.2)	4.4	(0.2)	4.0	(1.0)
**21–24**	**Total**	18.0	(0.1)	72.5	(2.2)	39.1	(2.0)	16.5	(1.1)	3.5	(0.1)	4.2	(0.1)	4.6	(0.6)
	Men	17.7	(0.1)	91.8	(4.0)	51.0	(3.6)	22.5	(1.9)	4.2	(0.1)	5.1	(0.1)	4.8	(0.7)
	Women	18.2	(0.1)	52.4	(2.4)	26.4	(2.0)	10.3	(1.1)	2.8	(0.1)	3.3	(0.1)	4.4	(1.0)
	White	17.9	(0.1)	77.0	(2.8)	43.5	(2.6)	17.2	(1.3)	3.6	(0.1)	4.2	(0.1)	5.1	(0.8)
	Black	18.6	(0.1)	68.4	(5.0)	23.5	(3.1)	12.0	(3.0)	2.7	(0.1)	3.8	(0.2)	2.5	(1.0)
	AIAN	14.8	(1.0)	74.0	(18.5)	44.3	(17.0)	40.2	(17.7)	5.7	(1.2)	3.7	(0.4)	3.2	(1.4)
	Asian/NHOPI	17.9	(0.2)	57.3	(7.8)	30.3	(6.1)	15.9	(5.4)	3.1	(0.3)	4.3	(0.5)	6.3	(2.7)
	Hispanic	18.2	(0.1)	60.1	(4.8)	34.0	(4.7)	15.4	(3.6)	3.7	(0.2)	5.0	(0.3)	4.0	(1.1)
	College, full-time	17.9	(0.1)	79.7	(4.5)	47.4	(4.0)	21.3	(2.1)	3.6	(0.1)	4.0	(0.1)	4.8	(1.0)
	College, part-time	18.3	(0.2)	60.5	(6.2)	33.2	(5.5)	16.0	(3.2)	3.5	(0.3)	4.1	(0.3)	3.2	(1.1)
	Noncollege	17.9	(0.1)	70.9	(2.8)	35.8	(2.4)	14.2	(1.4)	3.5	(0.1)	4.4	(0.1)	4.8	(0.9)

1 Calculation included former drinkers

2 SE = standard error

3 AIAN = American Indian/Alaska Native

4 NHOPI = Native Hawaiian/Other Pacific Islander

**Table 3:** Percentage Distribution of Usual Drinking Location by Beverage Type Among Young Adult Current Drinkers Ages 18–20 and 21–24, According to Sex, Race/Ethnicity, and College Enrollment Status, NESARC, 2001–2002

Beverage	Sex, Race/Ethnicity, College Enrollment Status	In Own Home				In Homes of Friends or Relatives				In Public Places			

		Ages 18–20		Ages 21–24		Ages 18–20		Ages 21–24		Ages 18–20		Ages 21–24	
		%	(SE)^1^	%	(SE)	%	(SE)	%	(SE)	%	(SE)	%	(SE)
**Coolers**	**Total**	31.1	(2.3)	37.8	(1.8)	59.1	(2.3)	33.0	(1.8)	9.8	(1.3)	29.2	(1.7)
	Men	26.1	(3.0)	37.6	(2.8)	62.7	(3.4)	37.6	(3.1)	11.2	(2.3)	24.8	(2.9)
	Women	34.6	(3.2)	38.0	(2.3)	56.6	(3.3)	29.7	(1.9)	8.8	(1.6)	32.3	(2.0)
	White	31.6	(2.8)	35.6	(2.3)	59.2	(2.9)	35.0	(2.3)	9.2	(1.6)	29.4	(2.3)
	Black	30.3	(5.3)	43.2	(4.4)	60.1	(5.7)	28.4	(4.1)	9.6	(3.0)	28.4	(3.9)
	AIAN^2^	28.1	(17.8)	66.1	(11.8)	57.1	(18.3)	19.4	(9.7)	14.8	(13.5)	14.5	(9.5)
	Asian/NHOPI^3^	21.9	(10.1)	39.0	(6.5)	66.7	(11.9)	25.8	(6.3)	11.4	(8.0)	35.2	(7.2)
	Hispanic	31.9	(5.8)	39.6	(4.6)	55.3	(6.8)	31.8	(4.1)	12.8	(3.6)	28.6	(4.1)
	College, full-time	33.7	(4.3)	29.5	(3.2)	54.5	(4.5)	37.2	(3.1)	11.9	(2.5)	33.3	(2.7)
	College, part-time	35.2	(10.7)	40.6	(5.8)	64.2	(10.7)	29.1	(6.0)	0.6	(0.6)	30.3	(4.9)
	Noncollege	29.5	(2.6)	42.6	(2.3)	61.1	(2.9)	31.1	(2.2)	9.4	(1.7)	26.3	(2.1)
**Beer**	**Total**	26.8	(1.7)	37.0	(1.8)	62.0	(1.9)	25.7	(1.4)	11.1	(1.3)	37.4	(1.8)
	Men	29.0	(2.2)	41.2	(2.3)	61.0	(2.3)	29.9	(2.0)	9.9	(1.5)	28.9	(2.2)
	Women	23.1	(2.5)	30.2	(2.3)	63.8	(3.0)	19.0	(1.8)	13.2	(2.3)	50.7	(2.5)
	White	23.8	(2.0)	33.1	(2.0)	65.3	(2.3)	23.8	(1.6)	10.9	(1.6)	43.0	(2.1)
	Black	29.7	(5.2)	41.5	(4.7)	54.5	(5.6)	34.0	(4.6)	15.7	(4.5)	24.5	(3.7)
	AIAN	14.6	(7.9)	25.9	(10.4)	85.4	(7.9)	27.0	(10.7)	0.0	(0.0)	47.1	(11.7)
	Asian/NHOPI	31.1	(9.6)	39.3	(7.7)	51.4	(10.4)	31.5	(6.3)	17.4	(8.7)	29.2	(6.0)
	Hispanic	40.1	(3.8)	51.4	(3.3)	50.3	(3.7)	26.8	(3.0)	9.6	(2.2)	21.8	(2.6)
	College, full-time	28.6	(3.3)	27.3	(3.1)	60.7	(3.5)	24.5	(2.3)	10.7	(2.3)	48.3	(3.0)
	College, part-time	33.5	(7.3)	34.6	(5.0)	57.6	(8.9)	24.5	(4.1)	8.9	(3.8)	40.9	(4.9)
	Noncollege	25.5	(1.8)	42.8	(2.0)	63.0	(2.2)	26.5	(1.9)	11.5	(1.6)	30.7	(1.9)
**Wine**	**Total**	38.9	(3.0)	39.0	(1.9)	43.0	(2.8)	31.0	(1.6)	18.0	(2.1)	30.0	(1.8)
	Men	36.9	(4.1)	34.9	(2.8)	47.3	(4.2)	34.1	(2.7)	15.8	(2.7)	31.1	(2.9)
	Women	41.0	(4.2)	42.2	(2.4)	38.7	(3.8)	28.7	(2.0)	20.3	(3.0)	29.2	(2.3)
	White	38.2	(3.5)	40.9	(2.3)	43.7	(3.2)	30.0	(1.8)	18.1	(2.5)	29.1	(2.0)
	Black	25.5	(7.8)	31.8	(5.1)	47.3	(10.0)	23.8	(4.9)	27.2	(9.4)	44.4	(6.4)
	AIAN	60.2	(16.4)	75.2	(15.1)	32.3	(15.5)	7.5	(7.5)	7.5	(7.4)	17.3	(13.8)
	Asian/NHOPI	32.3	(22.2)	26.8	(6.8)	67.7	(22.2)	46.9	(8.2)	0.0	(0.0)	26.3	(7.3)
	Hispanic	47.4	(7.3)	35.4	(5.2)	35.9	(6.6)	36.6	(5.0)	16.7	(4.2)	28.0	(4.3)
	College, full-time	38.0	(5.2)	39.8	(3.0)	40.9	(5.1)	30.8	(2.9)	21.1	(4.1)	29.4	(3.3)
	College, part-time	42.8	(11.6)	37.2	(5.4)	40.3	(12.0)	34.7	(5.1)	16.8	(7.0)	28.0	(4.5)
	Noncollege	39.1	(4.0)	38.8	(2.9)	44.5	(3.7)	30.2	(2.6)	16.4	(2.4)	31.0	(2.5)
**Liquor**	**Total**	23.3	(1.8)	20.5	(1.5)	61.8	(1.9)	22.4	(1.5)	14.8	(1.7)	57.1	(1.9)
	Men	22.1	(2.4)	22.7	(2.2)	65.0	(2.7)	29.4	(2.4)	12.9	(2.0)	47.9	(2.8)
	Women	24.8	(2.8)	18.3	(1.9)	57.9	(3.2)	15.6	(1.6)	17.2	(2.7)	66.0	(2.2)
	White	24.5	(2.2)	20.0	(1.8)	62.4	(2.3)	19.3	(1.6)	13.0	(2.0)	60.8	(2.2)
	Black	21.5	(4.9)	20.5	(3.0)	57.1	(6.2)	34.5	(4.7)	21.3	(5.3)	45.0	(4.6)
	AIAN	28.2	(13.2)	9.9	(6.1)	47.2	(13.3)	11.6	(8.4)	24.6	(12.2)	78.4	(9.7)
	Asian/NHOPI	8.5	(6.6)	17.7	(6.0)	70.7	(12.4)	31.4	(8.8)	20.9	(11.2)	50.9	(6.7)
	Hispanic	19.5	(4.7)	27.0	(3.7)	62.7	(6.1)	29.7	(3.5)	17.8	(4.4)	43.3	(4.0)
	College, full-time	27.2	(3.3)	14.6	(2.3)	56.6	(3.6)	21.9	(2.5)	16.1	(3.1)	63.5	(3.3)
	College, part-time	27.0	(9.3)	20.7	(4.0)	53.5	(10.3)	20.3	(3.9)	19.5	(7.5)	59.0	(5.5)
	Noncollege	21.0	(2.0)	24.5	(2.0)	65.2	(2.3)	23.3	(2.0)	13.8	(2.0)	52.2	(2.3)

1 SE = standard error

2 AIAN = American Indian/Alaska Native

3 NHOPI = Native Hawaiian/Other Pacific Islander

## Footnotes

1 More information on computational details of the data analysis as well as access to public use files of the data are available at http://niaaa.census.gov/.

2 Reported frequencies of drinking were recorded by the survey in a multiple-choice format, with the options being: every day, nearly every day, 3 to 4 times a week, 2 times a week, once a week, 2 to 3 times a month, once a month, 7 to 11 times in the last year, 3 to 6 times in the last year, and 1 or 2 times in the last year. Using the midpoints of the categorical response options, the frequencies were converted to number of drinking days per year to calculate the means.

3 Statistically significant differences can be roughly assessed by the nonoverlapping 95-percent confidence intervals (i.e., the estimates plus and minus two times their standard errors).

4 The relatively large standard errors of these estimates result in part from the small sample size of this group.

5 The NSDUH public-use data include the following age groups: ages 12, 13, … 21, 22–23, 24–25, 26–29,30–34, 35–49, 50–64, and 65 and older.
